# Case Report: A rare case of bilateral molar natal teeth in a term newborn

**DOI:** 10.3389/fdmed.2024.1336865

**Published:** 2024-04-25

**Authors:** B. M. Varriano, L. Ades, S. R. Vaughan

**Affiliations:** Division of Neonatology and Newborn Medicine, Department of Pediatrics, Massachusetts General Hospital and Harvard Medical School, Boston, MA, United States

**Keywords:** decision-making, cranio-maxillofacial surgery, prevention, risk factor(s), tooth development, natal teeth

## Abstract

Reports of natal and neonatal teeth have been well documented in the literature, although the presence of natal primary molars remains rare, and a clear management strategy does not exist. This is a case study of a female newborn delivered at a gestational age of 41 weeks 1 day to a 31-year-old G1P1001 mother. Apgar scores were 8 and 9 at 1 and 5 min, respectively. The infant was delivered by cesarean section and was admitted to the Mass General Hospital Newborn Nursery, where she received routine care. The patient had two posterior molars, which warranted consultation for oral maxillofacial surgery. Due to the gross mobility of the natal teeth and the risk of aspiration in a small breastfeeding newborn, the decision was made to extract the natal teeth immediately. Understanding the management of natal teeth is important for the pediatrician.

## Background

Reports of natal and neonatal teeth have been well documented in the literature in the last 70 years, although the presence of natal primary molars remains rare. Natal teeth are those that are already present at birth and neonatal teeth are those that erupt during the first 30 days of birth and are biologically normal. Natal teeth are reported to be more abundant than neonatal teeth (1, 2). Natal teeth, when present, present as mandibular primary incisors 85% of the time, maxillary incisors 11% of the time, and posterior teeth 4% of the time (3).

The etiology of natal and neonatal teeth continues to remain unknown despite suggestive causative factors, including maternal exposure to environmental toxins, hypovitaminosis, infection, endocrinal disturbances, and hereditary transmission of an autosomal dominant gene (4–8). In some studies, natal or neonatal molars identified may be associated with systemic conditions or syndromes (i.e., histiocytosis X, cleft lip and palate, Pfeiffer syndrome); however, there are no current studies that directly link a causative relationship between natal teeth and syndromes (9–11). Common presentations of natal teeth that warrant further evaluation include tooth mobility, difficulty with breast latch or suckle, and concern for aspiration (12–14). Ulceration of the ventral surface of the tongue, also known as Riga–Fede disease, is a known complication that can occur in the presence of natal or neonatal teeth, and thus, it is important that these are evaluated upon initial observation (14–16). There is controversy over the prevalence of occurrence of natal and neonatal teeth in male and female infants. Some systematic reviews have reported no significant difference between male and female infants in tooth morphology (1, 2, 17); however, other authors report a higher occurrence of natal teeth in female newborns compared to male newborns (2, 18).

In this study, we present a noteworthy case of a 2-day-old infant who was born with two maxillary posterior molar natal teeth. According to the American Academy of Pediatric Dentistry, the average age for primary maxillary first molars to erupt is between 11 and 18 months (19, 20). The two teeth in this case may represent the premature eruption of maxillary primary molars, which is a rare finding in newborns.

## Case

### Birth summary

A female newborn was delivered at a gestational age of 41 weeks 1 day to a 31-year-old G1P1001 mother. The Apgar scores were 8 and 9 at 1 and 5 min, respectively. The infant was delivered by cesarean section due to concerns of category 2 tracing and fetal intolerance to labor. The physical examination at the time of birth was unremarkable. The newborn patient received vitamin K IM and erythromycin ointment at the time of birth. The patient was admitted to the Mass General Hospital Newborn Nursery, where she received routine care. Routine newborn screenings (metabolic, hearing, bilirubin, cardiac) were performed for 36 h after birth. Both parents consented to Hepatitis B vaccination. The newborn weighed 3,070 g (12.61 percentile) and was 50.8 cm in length (38.87 percentile). Her head circumference was not measured at the time of birth. The only imaging before birth was performed during the first trimester.

### Family history

Family history was negative for hyperbilirubinemia in the newborn, congenital heart disease, childhood kidney disease, and developmental dysplasia of the hip. The mother had a history of acquired left-sided partial hearing loss. There was no family history of dental or bone abnormalities.

**Figure 1:**
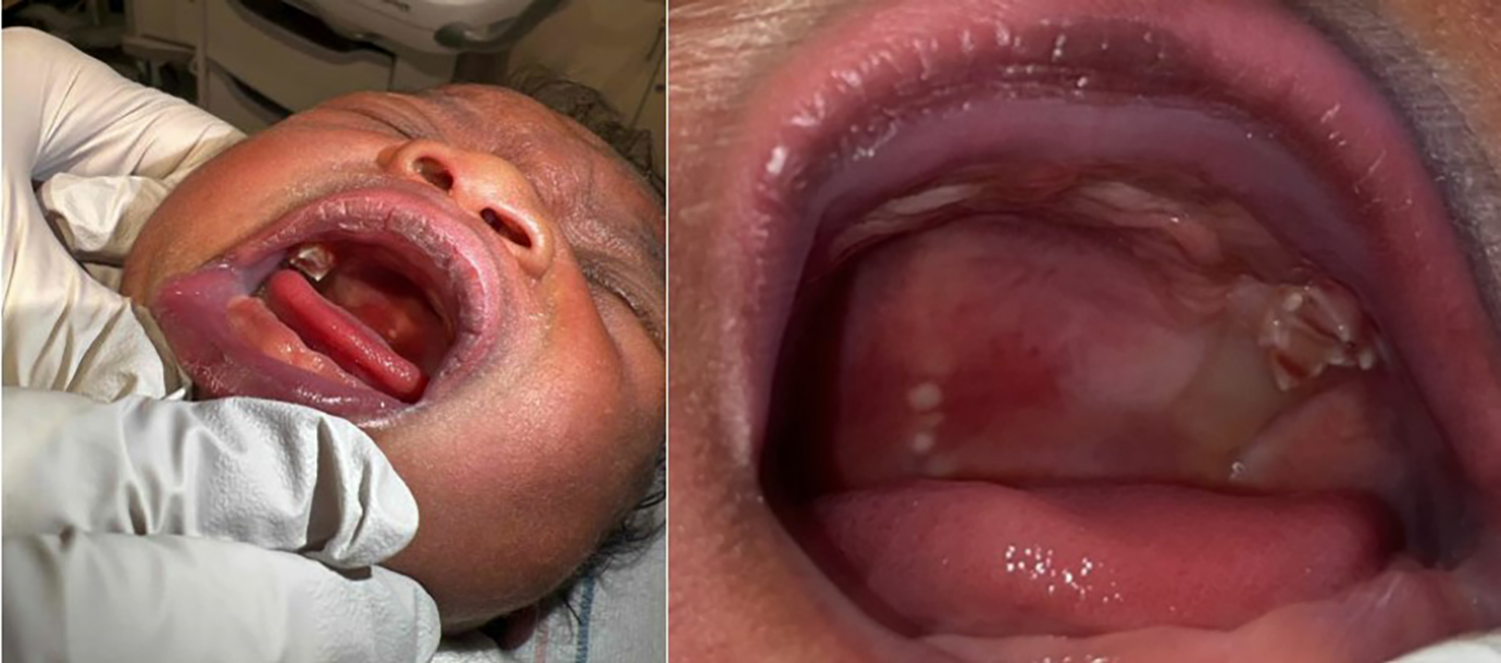
Intraoral examination revealed the white crowns of two erupted primary maxillary first molars. Natal tooth B (Left image) was fully erupted with class III mobility. Natal tooth I (Right image) was partially erupted with class III mobility. Of note, the newborn has Ebstein Pearls, on the midline of the soft palate.

### Oral maxillofacial surgery management

Oral and maxillofacial surgery was consulted. A discussion of the risks and benefits of not removing the teeth was had before the parents chose to remove the teeth. Upon an extraoral examination, the newborn appeared otherwise healthy and non-syndromic. An intraoral examination revealed the white crowns of two erupted primary maxillary first molars. Natal teeth #B and #I presented with sharp cusp tips, thin fragile enamel, and grade III mobility (see Figure 1). Due to the mobility of the teeth, the mother had difficulties with feeding and latching. Ultimately, given the difficulty in feeding and future risk of aspiration, the parents opted to have the teeth removed. The teeth were removed using oral glucose and benzocaine topical anesthesia paste with a curved snap. Of note, the crown was removed without evidence of root formation. Hemostasis was easily achieved after extraction and the newborn patient tolerated the procedure well.

## Discussion

The presence of erupted teeth at birth or the eruption of teeth within 30 days after birth is considered a rare occurrence, with an incidence rate of 0.1% (20). In this case of a 2-day-old female infant delivered via cesarean section at 41 weeks of gestation, with no abnormalities detected at routine pregnancy screenings, the presence of two fully formed maxillary molar teeth were noted at birth.

According to the American Academy of Pediatric Dentistry, the average age for primary maxillary first molars to erupt is between 11 and 18 months (19, 20). In this study, maxillary molars were present from birth. The case of this 2-day-old infant is noteworthy in that the location of the two natal teeth was in the maxillary primary molar region whereas the majority of cases of natal teeth described in the literature are incisors (21). The management of incisors and molars varies. Incisors are often removed since there is little risk of removing them. The management of molars, however, varies depending on tooth maturity. Immature molars should be removed, as they are less likely to develop normally. However, mature molars should not be removed. Maturity is typically identified via radiographs or histology. However, the mobility of the teeth can also give a good estimation of the level of maturity of the teeth. The teeth in our study are most likely immature, given the high degree of mobility. Mature teeth, on the other hand, do not typically present with mobility. Lastly, this newborn was born with two molars. This is consistent with the other literature, where the average number of natal molars is one or two (21). One of the highest numbers of natal molars was documented in a study, where the baby was born with eight natal molars, four in the maxillary site and four in the mandibular site (22). In that study of eight natal molars, the patient required resuscitation, which was not an issue for our patient, likely given the number of teeth.

In some studies, natal or neonatal molars have been suggested to be associated with systemic conditions or syndromes (i.e., histiocytosis X); however, there are no current studies that directly link a causative relationship between natal teeth and syndromes (9). There are some disorders associated with natal teeth, but given the rarity, there are no conditions that are highly linked with molars specifically. In addition to systemic conditions and developmental disorders, natal teeth have been associated with the congenital defect known as cleft lip and palate, which is inherited in a multifactorial manner (23, 24). Currently, there is no known etiology of natal teeth. Some theories suggest that the development of natal teeth is a response to febrile states, endocrine disturbances, nutritional deficiencies, or a response to congenital syphilis (23). None of the above scenarios were present in this case, given our extent of knowledge of maternal health during gestation.

Finally, it is important to note that the management of natal teeth must be approached on a case-by-case basis. Erupted natal teeth may represent premature primary dentition. For this reason, they should be preserved with close monitoring by a pediatric dentist and pediatrician to ensure that they do not become an aspiration risk or impair feeding (20). However, in the present case, grade III mobility of the erupted teeth suggested that the natal teeth were immature and that not removing the teeth posed an increased risk for aspiration, especially given the small diameter of the infant airway. Ultimately, there was a decision by the parents of the newborn to remove the teeth. In our case, the teeth were causing issues with latching during breastfeeding, ultimately compromising fetal nutrition. Although this newborn did not present with ulcerations or trauma to the tongue, if the teeth remained in the mouth, there could be trauma to the adjacent soft tissue. Other complications of natal teeth can include pain and lacerations of the mother's breast, and the potential risk for hemorrhage, particularly in the setting of vitamin K deficiency (20).

Hemorrhage often occurs at the time of extraction and is a rarer occurrence nowadays, given current practices. Now, before teeth extraction, a consultation with a pediatrician is recommended to make sure that the newborn has received vitamin K prophylaxis after birth (20). Previously, a physician would wait until the 10th day for tooth extraction to prevent excessive hemorrhage from occurring. However, today, newborns are routinely given 1.0 mg of vitamin K after birth, which eliminates the need to wait until the 10th day (21). In addition, the removal of a natal tooth may impact the future development of primary and permanent dentition. Monitoring by a pediatric dentist and oral surgeon is important to ensure the normal development of primary and permanent dentition and to exclude any other dental abnormalities.

## Conclusion

Understanding the management of natal teeth is important for the pediatrician. While the cause of natal teeth is not fully understood, the positioning of the teeth can determine the need for removal, especially if the teeth are immature. In the case of a maxillary molar with grade III mobility, as reported in the newborn in this study, known complications such as problems with breastfeeding are common; therefore, the decision was made to remove the teeth.

## Data Availability

The original contributions presented in the study are included in the article/Supplementary Material, and further inquiries can be directed to the corresponding author.
